# Examining the associations between mental health, life balance, work-method autonomy, and perceived boundary control among postdoctoral fellows

**DOI:** 10.3389/fpsyg.2024.1416724

**Published:** 2024-12-19

**Authors:** Brian K. Lo, In Young Park, David Choung, Melissa McTernan, Erin Sibley, Kirsten K. Davison

**Affiliations:** ^1^Department of Family Relations and Applied Nutrition, University of Guelph, Guelph, ON, Canada; ^2^School of Social Work, Boston College, Chestnut Hill, MA, United States; ^3^Morrissey College of Arts and Sciences, Boston College, Chestnut Hill, MA, United States; ^4^Research Services, Boston College, Chestnut Hill, MA, United States; ^5^Office of the Vice Provost for Research, Boston College, Chestnut Hill, MA, United States

**Keywords:** postdoctoral fellow, mental health, life balance, work autonomy, anxiety, depressive disorder symptoms

## Abstract

High mental health risks and life imbalance among postdoctoral fellows (postdocs) are persistent concerns in academia. However, little is known about the relationship between these two subjects and whether autonomy at work is linked to life balance among postdocs. Online survey responses from 117 postdocs (59% women; 49% non-Hispanic white) were assessed using multiple linear regression analysis to examine whether the work-method autonomy and perceived boundary control of postdocs were linked to life balance. Additionally, logistic regression analysis was used to examine whether postdocs who reported better life balance had lower risks of reporting mood disorder symptoms. We found that 39%, 27%, and 45% of postdocs reported anxiety, depressive, and anxiety-or-depressive disorder symptoms, respectively. Both work-method autonomy and perceived boundary control were positively associated with life balance [*B* = 0.40, 95% CI = [0.20–0.60]; *B* = 0.50, 95% CI = [0.32–0.67], respectively]. Postdocs with greater life balance had decreased odds of reporting mood disorder symptoms [anxiety disorder symptoms: adjusted OR = 0.55, 95% CI = (0.37–0.82); depressive disorder symptoms: adjusted OR = 0.31, 95% CI = (0.18–0.55); anxiety-or-depressive disorder symptoms: adjusted OR = 0.42, 95% CI = (0.27–0.65)]. Postdocs' mental health appeared to be influenced by life balance, which may be enhanced by providing work-method autonomy and increasing perceived boundary control.

## 1 Introduction

In the United States (US), postdoctoral fellows (postdocs) are an essential component of the academic workforce, with at least 60,000–100,000 working in various fields according to national surveys (National Academy of Sciences, National Academy of Engineering, and Institute of Medicine, 2014; National Center for Science Engineering Statistics, 2024). Postdocs are skilled professionals who hold doctoral degrees and provide research or teaching support in exchange for training and mentorship to prepare themselves for their chosen career paths. Their contributions to scientific knowledge and teaching innovation include grant writing, project management, data analysis, and providing mentorship to junior researchers and students. However, the substantial contributions of postdocs do not come without a price. Low salaries, long hours, and high job strain are persistent training environment concerns reported by postdocs, raising serious mental health concerns (Udesky, 2023; Woolston, 2020a).

Despite recent increases in fellowship compensations from the US National Institutes of Health (NIH), postdoc salaries in academia remain considerably lower than those in government, industry, or non-profit sectors (Morin et al., 2022; Heidt, 2024). For example, US research scientists with training similar to postdocs earn a median salary of $136,000 USD (Franklin University, n.d.), almost 50% higher than the NIH-reported range for postdocs ($54,000–74,000 USD, depending on experience) (Heidt, 2024). Most postdocs in the US earn at or below the national median household income of US$74,680 (US Census Bureau, 2023). Other countries such as Australia, Canada, and New Zealand also report postdocs earning below or just above their national averages (Australian Bureau of Statistics, 2024; Canadian Association of Postdoctoral Scholars, 2023; Seek, 2024; Statistics Canada, 2023; Stats NZ, 2023; PayScale, 2023; Trager, 2024).

These financial hardships are compounded by long working hours, which postdocs report as a common experience. In a global survey of over 7,000 postdocs, nearly half reported a culture of long hours, with many working beyond contracted hours and on days off or weekends (Woolston, 2020a). In the US, some data showed that postdocs work an average of 53 h per week, compared to the national average of 34.4 h (US Bureau of Labor Statistics, 2024; Stephan, 2013). This trend is mirrored in other countries. An Australian study of 280 postdocs found that over 75% worked more than 41 h per week, with 20% working more than 50 h (Hardy et al., 2016). Similarly, nearly half of Canadian postdocs in a national survey reported working more than 40 h per week, with 24% working between 50 and 59 h, well-above the national average of 36 h per week for Canadians (Jadavji et al., 2016; Statistics Canada, 2023).

The combination of financial strain and excessive work hours is exacerbated by the intensely competitive nature of academia, where postdocs compete for highly sought-after permanent positions, such as faculty roles (McConnell et al., 2018; Woolston, 2020a; Pitt et al., 2021). This resulting stress is further compounded by inadequate support from supervisors, which has been linked to poor mental health outcomes (Pitt et al., 2021; Morin et al., 2022; van der Weijden and Teelken, 2023). Additionally, precarious job prospects and unclear career trajectories can contribute to postdocs' strain, negatively impacting their wellbeing (van der Weijden and Teelken, 2023; Nicholls et al., 2022; Udesky, 2023; van Benthem et al., 2020).

Despite these documented challenges, research on postdoc workplace behaviors and mental health has been overlooked. Currently, there is no official reporting of postdocs' mental health status in the US. However, existing non-governmental statistics shed light on the high prevalence of mental health issues among postdocs. For instance, an international postdoc survey conducted by *Nature* with over 7,600 respondents revealed that 51% of surveyed postdocs had considered leaving research due to work-related mental health concerns (Woolston, 2020b). A US-based study involving 6,292 postdocs found that postdocs experienced elevated stress during the COVID-19 pandemic, with primary stressors being a combination of work, family, and emotional burdens (Morin et al., 2022). The lack of official reporting on postdoc mental health may be due to institutional disinterest, as postdocs are viewed as temporary or transitional labor. Furthermore, the scientific community tends to prioritize postdoc productivity and career outcomes over health and wellbeing (Woolston, 2020b). In fact, postdocs may be more vulnerable to mental health challenges than other academics, as their status often limits their access to or full benefit from institutional mental health support available to full-time students and faculty (Nicholls et al., 2022; Morin et al., 2022).

Given the lack of mental health research among postdocs and the critical role of work–life balance in their decision to remain in academia (Ysseldyk et al., 2019; Morin et al., 2022), it is essential to identify ways to foster balance across all aspects of postdocs' life domains and mental wellbeing. In the present study, we chose to focus on *life balance* rather than *work–life balance*, as life balance is a broader concept, defined as “*individuals' global perceptions of alignment between their actual involvement in all life domains of personal relevance, and their personal needs*” (Reinke and Gerlach, 2022). Life balance acknowledges that individuals engage with multiple life domains beyond work and family, such as friends, hobbies, health, and wellness, and that the importance of these domains can vary over time and across individuals (Casper et al., 2018). The life balance concept also accounts for how individuals assess whether their participation in various life domains aligns with their personal needs (Reinke and Gerlach, 2022).

According to Self-Determination Theory, optimal functioning and wellbeing are achieved when individuals' needs for autonomy, competence, and relatedness are met (Deci and Ryan, 2000). Autonomy is central to achieving life balance because it allows individuals to feel in control of their actions and to align them with personal needs (Deci and Ryan, 2000). Thus, providing postdocs with the independence to decide how to carry out work tasks (known as *work-method autonomy*) (Morgeson and Humphrey, 2006) may enhance their performance and efficiency, allowing them to allocate time across various life domains as desired, which supports life balance (de Vargas Pinto et al., 2023). Additionally, postdocs' ability to control the frequency and timing with which they structure work and non-work life to meet their roles and demands (known as *perceived boundary control*) (Kossek et al., 2012) may also support life balance. This control allows postdocs to focus on the life domain(s) they are currently engaged in, reducing distractions, and fostering alignment between personal needs and actual involvement (Ashforth et al., 2000; Reinke and Gerlach, 2022).

The Person-Environment Fit Theory posits that individuals and their environments influence each other, with a match between personal needs and the supplies in the environment (e.g., intrinsic rewards derived from involving in desired activities or experiences) leading to positive outcomes (Edwards et al., 1998; Edwards and Rothbard, 1999). Perceptions of life balance arise from comparing personal needs to the actual participation in significant life domains in the environment (Reinke and Gerlach, 2022). That is, when postdocs experience a mismatch between their personal needs and the rewards they derive from involvement in various life domains in the environment, this “low life balance” can lead to tension, dissatisfaction, and negative impacts on mental health, and vice versa (Edwards and Rothbard, 1999; Edwards et al., 1998).

To date, no studies have examined the links between postdocs' work-method autonomy/perceived boundary control and their life balance. Only two studies have explored connections between work–life balance and mental health among postdocs (Ysseldyk et al., 2019; Pitt et al., 2021). However, as discussed, a work–life balance perspective may overlook essential life domains beyond work and family (Reinke and Gerlach, 2022). This study aims to address these gaps by examining the relationships between work-method autonomy/perceived boundary control and life balance, as well as between life balance and mood disorder symptoms (i.e., anxiety and depression) among postdocs.

Specifically, we hypothesized the following:

Hypothesis 1: Postdocs' work-method autonomy and perceived boundary control are positively linked to life balance.

Hypothesis 2: Greater life balance is associated with lower levels of mood disorder symptoms (anxiety- and depressive-disorder) among postdocs.

## 2 Methods

### 2.1 Data collection procedures

The present study took place in Massachusetts, a state in the US with major research institutions, such as Harvard University and Massachusetts Institute of Technology, and with one of the largest postdoc populations in the country (PostdocInUSA, 2021). To be eligible, participants must be 18 years and older, held a postdoc appointment at an institution in Massachusetts at the time of participation, and were able to complete an online survey in English. Because there is no comprehensive contact list of all postdocs in Massachusetts for research, we used convenience sampling for recruitment by circulating a survey invitation through email distribution lists of postdocs and social media platforms. We used multiple strategies to compile a comprehensive emailing list for sending out our survey invitation. First, we identified 110 higher education institutions in Massachusetts through the Carnegie Classifications of Institutions of Higher Education database. For each institution, we determined through online searches if they had (1) an institutional Postdoctoral Office, which are run by professionals and funded by institutions, and (2) Postdoctoral Associations, which are largely managed by postdocs themselves. Email and twitter handles, if available, for both institutional groups were collected. If we could not identify an institution's Postdoctoral Office or Association, we collected the email address of the Human Resources office (HR); if HR's contacts were unavailable, Dean's Office or generic contacts were collected. We also contacted the Boston Postdoctoral Association, a local coalition of postdoc associations, to gather any contact information we missed based on the online search.

We emailed 12 institutional Postdoctoral Offices, 17 Postdoctoral Associations, and 87 non-postdoc offices to describe the study and ask them to distribute our survey link to their postdocs. The Boston College Office for Research Protections also posted the survey link on their X (formerly and colloquially known as Twitter) and used the @mention function to notify institutional Postdoctoral Offices and Associations about the study.

Data were collected online through a Qualtrics survey implemented between April and July 2022. To incentivize participation, participants were offered the opportunity to enter a draw to win one of twenty $50 USD Amazon gift cards. Participants included in the study indicated their informed consent and study activities were approved by the Boston College Institutional Review Board (IRB protocol# 22.223.01e; initial approval date: March 21, 2022).

### 2.2 Measures

**Anxiety disorder symptoms** and **depressive disorder symptoms** were measured using the Generalized Anxiety Disorder 2-item (GAD-2) (Kroenke et al., 2007) and Patient Health Questionnaire-2 (PHQ-2), respectively (Kroenke et al., 2003). GAD-2 and PHQ-2 are short versions of GAD 7-item (GAD-7) and PHQ 9-item (PHQ-9) and have been found reliable screening measures in practical and clinical settings (Staples et al., 2019). Each item in each scale asked postdocs to indicate their frequency of experiencing certain symptoms in the past 2 weeks: 0 = not at all, 1 = several days, 2 = more than half the days, and 3 = nearly every day. Items in GAD-2 were “*Feeling nervous, anxious or on edge*” and “*Not being able to stop or control worrying*.” Items in PHQ-2 were “*Feeling down, depressed or hopeless*” and “*Little interest or pleasure in doing things*.” Consistent with guidelines from the Centers for Disease Control, a simple sum score was calculated for each instrument, with a score of three or greater on the GAD-2 or the PHQ-2 indicative of anxiety or depressive disorders symptomology, respectively (National Center for Health Statistics, 2022). This cut-off is also associated with generalized anxiety disorder and major depressive disorder (National Center for Health Statistics, 2022). Based on this cut-off, we dichotomized participants into whether they reported anxiety, depressive, and anxiety-or-depressive disorder symptoms. For the present study, the Cronbach's alpha coefficients (α) for GAD-2 and PHQ-2 were 0.83 and 0.85, respectively.

**Life balance** was measured using the 4-item scale from Reinke and Gerlach (2022) that assesses one's global perception of alignment between their actual involvement in all life domains of personal relevance and their personal needs. Each item was rated on a 5-point Likert scale (1 = Strong disagree to 5 = Strongly agree). A sample item is: “*Overall, I was able to live my life in all domains just as I wished*.” Items were averaged to create a scale score, with higher scores reflecting greater life balance (study sample α = 0.94).

**Work-method autonomy**, or one's level of autonomy in choosing the methods they employ to finish work tasks, was measured using three items from Morgeson and Humphrey (2006) Work Design Questionnaire. Each item was rated on a 5-point Likert scale (1 = Strong disagree to 5 = Strongly agree). A sample item is: “*My postdoc position allows me to decide on my own how to go about doing my work*.” Items were averaged to create a scale score, with higher scores reflecting greater work-method autonomy (study sample α = 0.92).

**Perceived boundary control** was measured using the 3-item Boundary Control scale by Kossek et al. (2012) that assesses one's level of boundary control between work and personal life. Each item was rated on a 5-point Likert scale (1 = Strong disagree to 5 = Strongly agree). A sample item is: “*I control whether I am able to keep my work and personal life separate*.” Items were averaged to create a scale score, with higher scores reflecting greater perceived boundary control (study sample α = 0.88).

**Demographic characteristics** included age, race/ethnicity, gender identity, annual postdoc income, marital status, parental status, discipline, and immigration status.

### 2.3 Statistical analysis

Descriptive statistics (percentages/counts) were used to characterize postdocs' demographics and mood disorders symptoms. We used Fisher's exact tests to examine if mental health outcomes differed by postdocs' demographic characteristics. Fisher's exact test is appropriate when the expected count in table cells are small (i.e., <5) (Mehta and Patel, 2011, p. 1; Bland, 2015, p. 197). This is the case of our data that we have smaller sample size for certain demographic groups such as non-Hispanic black and other gender identities. All socioeconomic variables were modeled as categorical data.

To test Hypothesis 1, separate multiple linear regression models were used to examine the associations between life balance and the independent variables: work-method autonomy and perceived boundary control. We controlled for postdocs' age, gender identity, and race/ethnicity as these are associated with one's mental health status based on national data (Newall and Machi, 2020).

To test Hypothesis 2, we conducted a logistic regression analysis to assess the association between postdocs' life balance and the odds of reporting anxiety disorder symptoms, depressive disorder symptoms and anxiety-or-depressive disorder symptoms. Each outcome was tested in a separate regression model. Unadjusted and adjusted models were fit. In the latter, we controlled for the same covariates as in testing Hypothesis 1.

Data were cleaned and analyzed using SAS version 9.4 software (SAS institute Inc., Cary, NC, USA) and Stata version 17.0. There were 245 respondents. We excluded surveys that were not submitted by the respondents (*n* = 32), respondents that did not meet the inclusion criteria (*n* = 57; five were not completing a postdoc training; 26 were not completing a postdoctoral in Massachusetts, and 26 did not hold a doctoral degree), and those deemed to be fraudulent based on published guidelines (Wang et al., 2023) (*n* = 39; 13 provided non-Massachusetts institution names, and 26 had multiple indicators of fraudulent responses, including unreasonably short completion times, conflicting information, and/or unrealistic information). This process resulted in a final analytical sample of 117 responses.

## 3 Results

Among the 117 participating postdocs, they were from intuitions in the Boston-Cambridge-Newton, Massachusetts census division, most were between 30 and 39 years old (*n* = 85; 72.7%; mean = 32.6; standard deviation = 3.72; minimum = 26 years old; maximum = 48 years old; skewness = 1.18; kurtosis = 2.04), and did not have a child (*n* = 93; 79.5%) (Table 1). Slightly more than half were women (*n* = 69; 59.0%), had an annual postdoc income between $50,000 and 59,999 USD (*n* = 77; 66.3%), were married/partnered (*n* = 75; 64.1%), and were international trainees with a work visa (*n* = 62; 53.0%). About half were in the field of life sciences or medicine alone (*n* = 57; 48.8%). About half of the postdocs were white (*n* = 57; 49.1%) followed by Asian (*n* = 33; 28.5%), Hispanic (*n* = 22; 19.0%), and other races/ethnicities (*n* = 4; 3.5%).

**Table 1:** Postdoctoral fellows' characteristics (*n* = 117).

**Sample characteristics**		
	***n***	**%**
**Age (years)**		
18–29	26	22.2
30–39	85	72.7
40 or older	6	5.1
**Race/ethnicity**		
Hispanic	22	19.0
Non-Hispanic, Asian	33	28.5
Non-Hispanic, black	1	0.9
Non-Hispanic, white	57	49.1
Non-Hispanic, other races or multiple races	3	2.6
Missing	1	–
**Gender identity**		
Man	43	36.8
Woman	69	59.0
Other identities	5	4.3
**Annual postdoc income (USD)**		
< $50,000	7	6.0
$50,000–54,999	41	35.3
$55,000–59,999	36	31.0
$60,000–69,999	23	19.8
$70,000 or more	9	7.8
Missing	1	–
**Marital status**		
Single	42	35.9
Partnered or married	75	64.1
**Parental status**		
A parent	24	20.5
Not a parent	93	79.5
**Discipline**		
Education	1	0.9
Environmental sciences	2	1.7
Engineering	2	1.7
Humanities	1	0.9
Life sciences	45	38.5
Medicine	12	10.3
Nursing	2	1.7
Physical sciences	6	5.1
Psychology	5	4.3
Social sciences	4	3.4
Others	3	2.6
Multidisciplinary	34	29.1
**Immigration status**		
US citizen or green card holder	55	47.0
Work visa holder (e.g., J1, F1, etc.)	62	53.0
**MOOD DISORDER SYMPTOMS**		
**Anxiety disorder symptoms** ^*^		
Yes	46	39.3
No	71	60.7
**Depressive disorder symptoms** ^*^		
Yes	31	26.5
No	86	73.5
**Either anxiety or depressive disorder symptoms** ^*^		
Yes	53	45.3
No	64	54.7

^*^Based on the ≥3 score cut-off of the Generalized Anxiety Disorder 2-item (GAD-2) for anxiety disorder symptoms and the Patient Health Questionnaire-2 (PHQ-2) for depressive disorder symptoms. To be considered of having either anxiety or depressive disorder symptoms, participants met either the ≥3 score cut-off of the GAD-2 or the PHQ-2.

All key variables, including GAD-2 score for anxiety disorder symptom, PHQ-2 score for depressive disorder symptom, work-method autonomy score, boundary control score and life balance score, were considered normally distributed by showing skewness and kurtosis less than the absolute values of 2 and 7, respectively (see Supplementary Table 1 for descriptive statistics) (Curran et al., 1996).

The correlation matrix of variables is presented in Supplementary Table 2. All of the correlations between variables were in the anticipated direction.

Of our sample, 39.3% reported anxiety disorder symptoms (*n* = 46), 26.5% reported depressive disorder symptoms (*n* = 31), and 45.3% reported anxiety-or-depressive disorder symptoms (*n* = 53). Postdocs' mental health did not differ by demographic characteristics, including their age, race/ethnicity, gender identity, annual postdoc income, marital status, parental status, discipline, and immigration status (Fisher's exact tests *p* > 0.05; Table 2). We also performed sensitivity analysis to examine if postdocs' mental health differed by white vs. non-white and by science, technology, engineering, and math (STEM) disciplines vs. non-STEM disciplines. No significant differences were observed.

**Table 2:** Counts and percentages of with and without reported mental health symptoms by characteristics (*n* = 117).

**Characteristics**	**Anxiety disorder symptoms**		*P*-value^*^	**Depressive disorder symptoms**		*P*-value^*^	**Either anxiety or depressive disorder symptoms**		*P*-value^*^
	**Yes** ***n*** **(%)**^∧^	**No** ***n*** **(%)**^∧^		**Yes** ***n*** **(%)**^∧^	**No** ***n*** **(%)**^∧^		**Yes** ***n*** **(%)**^∧^	**No** ***n*** **(%)**^∧^	
**Age**									
18–29	10 (38.5)	16 (61.5)	0.887	5 (19.2)	21 (80.8)	0.064	12 (46.2)	14 (53.8)	0.563
30–39	33 (38.8)	52 (61.2)			22 (25.9)		63 (74.1)			37 (43.5)	48 (56.5)	
40 or older	3 (50.0)	3 (50.0)			4 (66.7)		2 (33.3)			4 (66.7)	2 (33.3)	
**Race/ethnicity** ^a^									
Hispanic	9 (40.9)	13 (59.1)	0.807	8 (36.3)	14 (63.7)	0.673	11 (50.0)	11 (50.0)	0.296
Non-Hispanic, Asian	11 (33.3)	22 (66.7)			8 (24.2)		25 (75.8)			13 (39.4)	20 (60.6)	
Non-Hispanic, black	0 (0.0)	1 (100.0)			0 (0.0)		1 (100.0)			0 (0.0)	1 (100.0)	
Non-Hispanic, white	23 (40.4)	34 (59.6)			13 (22.8)		44 (77.2)			25 (43.9)	32 (56.1)	
Non-Hispanic, other races or multiple races	2 (66.7)	1 (33.3)			1 (33.3)		2 (66.7)			3 (100.0)	0 (0.0)	
**Gender identity**									
Man	14 (32.6)	29 (67.4)	0.372	11 (25.6)	32 (74.4)	0.238	18 (41.9)	25 (58.1)	0.722
Woman	29 (42.0)	40 (58.0)			17 (24.6)		52 (75.4)			32 (46.4)	37 (53.6)	
Other gender identity	3 (60.0)	2 (40.0)			3 (60.0)		2 (40.0)			3 (60.0)	2 (40.0)	
**Annual postdoc income (USD)** ^a^									
< $50,000	2 (28.6)	5 (71.4)	0.078	2 (28.6)	5 (71.4)	0.915	2 (28.6)	5 (71.4)	0.216
$50,000–54,999	23 (56.1)	18 (43.9)			13 (31.7)		28 (68.3)			24 (58.5)	17 (41.5)	
$55,000–59,999	9 (25.0)	27 (75.0)			8 (22.2)		28 (77.8)			12 (33.3)	24 (66.7)	
$60,000–69,999	9 (39.1)	14 (60.9)			6 (26.1)		17 (73.9)			11 (47.8)	12 (52.2)	
$70,000 or more	3 (33.3)	6 (66.7)			2 (22.2)		7 (77.8)			4 (44.4)	5 (55.6)	
**Marital status**									
Single	18 (42.9)	24 (57.1)	0.561	15 (35.7)	27 (64.3)	0.126	32 (42.7)	43 (57.3)	0.562
Partnered or married	28 (37.3)	47 (62.7)			16 (21.3)		59 (78.7)			21 (50.0)	21 (50.0)	
**Parent status**									
A parent	7 (29.2)	17 (70.8)	0.349	7 (29.2)	17 (70.8)	0.797	44 (47.3)	49 (52.7)	0.492
Not a parent	39 (41.9)	54 (58.1)			24 (25.8)		69 (74.2)			9 (37.5)	15 (62.5)	
**Discipline**									
Education	0 (0.0)	1 (100.0)	0.300	0 (0.0)	1 (100.0)	0.072	0 (0.0)	1 (100.0)	0.236
Environmental sciences	0 (0.0)	2 (100.0)			1 (50.0)		1 (50.0)			1 (50.0)	1 (50.0)	
Engineering	2 (100.0)	0 (0.0)			1 (50.0)		1 (50.0)			2 (100.0)	0 (0.0)	
Humanities	1 (100.0)	0 (0.0)			0 (0.0)		1 (100.0)			1 (100.0)	0 (0.0)	
Life sciences	16 (35.6)	29 (64.4)			9 (20.0)		36 (80.0)			17 (37.8)	28 (62.2)	
Medicine	7 (58.3)	5 (41.7)			7 (58.3)		5 (41.7)			9 (75.0)	3 (25.0)	
Nursing	2 (100.0)	0 (0.0)			2 (100.0)		0 (0.0)			2 (100.0)	0 (0.0)	
Physical sciences	1 (16.7)	5 (83.3)			1 (16.7)		5 (83.3)			2 (33.3)	4 (66.7)	
Psychology	2 (40.0)	3 (60.0)			0 (0.0)		5 (100.0)			2 (40.0)	3 (60.0)	
Social sciences	1 (25.0)	3 (75.0)			1 (25.0)		3 (75.0)			1 (25.0)	3 (75.0)	
Others	1 (33.3)	2 (66.7)		0 (0.0)	3 (100.0)		1 (33.3)	2 (66.7)	
Multidisciplinary	13 (38.2)	21 (61.8)		9 (26.5)	25 (73.5)		15 (44.2)	19 (55.8)	
**Immigration status**									
US citizen	19 (34.6)	36 (65.4)	0.348	14 (25.5)	41 (74.6)	0.837	23 (41.8)	32 (58.2)	0.577
Work visa holder	27 (43.6)	35 (56.4)			17 (27.4)		45 (72.6)			30 (48.4)	32 (51.6)	

^a^Numbers do not add up to 117 due to missing case (*n* = 1) under race/ethnicity and annual postdoc income.

^*^P-values from Fisher's exact test.

^∧^Row percentages.

The results for the multiple regression analysis predicting life balance are presented in Table 3. Both regression models, with work-method autonomy and perceived boundary control as main predictors, were statistically significant [*F*_(5,110)_ = 5.83, *p* < 0.001 *R*^2^ = 0.21; *F*_(5,110)_ = 9.37, *p* < 0.001, *R*^2^ = 0.30]. Both work-method autonomy and perceived boundary control were positively associated with life balance [*B* = 0.40, 95% CI = (0.20–0.60); *B* = 0.50, 95% CI = (0.32–0.67), respectively], after accounting for covariates. Thus, Hypothesis 1 was supported.

**Table 3:** Multiple regression analysis predicting life balance.

**Variable**	**Unstandardized coefficients**		**Standardized coefficients**	***t***	***p***	**VIF**	**DW**	**F(*df*)**	***R* ^2^**
	***B*** **(95% CI)**	***SE B***	β						
Work-method autonomy	0.40 (0.20, 0.60)	0.10	0.34	3.88	< 0.001	1.07	1.79	5.83(5)^***^	0.21
Perceived boundary control	0.50 (0.32, 0.67)	0.10	0.45	5.56	< 0.001	1.01	1.94	9.37(5)^***^	0.30

*B*, unstandardized beta; β, standardized beta; SE, standard error; CI, confidence intervals; VIF, variance inflation factor; DW, Durbin-Watson. Two independent variables (i.e., Work-method autonomy and perceived boundary control) were entered in the regression models separately; Covariates included age, gender identity, and race/ethnicity. Results for the covariates are omitted.

^***^p < 0.001.

In both the unadjusted and adjusted models, postdocs reporting higher life balance had significantly lower odds of reporting anxiety disorder symptoms [unadjusted OR = 0.53, 95% CI = (0.36–0.78); adjusted OR = 0.55, 95% CI = (0.37–0.82)], depressive disorder symptoms [unadjusted OR = 0.30, 95% CI = (0.18–0.52); adjusted OR = 0.31, 95% CI = (0.18–0.55)], and anxiety-or-depressive disorder symptoms [unadjusted OR = 0.42, 95% CI = (0.28–0.63); adjusted OR = 0.42, 95% CI = (0.27–0.65)] (Table 4). For every one-point increase in life balance score, there was a 45, 69, and 58% decrease in the odds of reporting anxiety disorder symptoms, depressive disorder symptoms, and anxiety-or-depressive disorders symptoms, respectively. Thus, Hypothesis 2 was also supported. We also performed sensitivity analyses to also control for participants' marital and parental statuses to account for the potential links with postdocs' mental health status in testing Hypothesis 2. The findings remained the same across all three adjusted models, showing significant associations between life balance and anxiety disorder symptoms [adjusted OR = 0.51, 95% CI = (0.34–0.78)], depressive disorder symptoms [adjusted OR = 0.29, 95% CI = (0.29–0.53)], and anxiety-or-depressive disorder symptoms [adjusted OR = 0.39, 95% CI = (0.25–0.62)], respectively.

**Table 4:** Associations between postdocs' life balance and mood disorder symptoms.

	**Anxiety disorder symptoms**			**Depressive disorder symptoms**			**Anxiety or depressive disorder symptoms**		
**Predictors**	**Odds ratio (95% CI)**	**SE**	χ^2^	**Odds ratio (95% CI)**	**SE**	χ^2^	**Odds ratio (95% CI)**	**SE**	χ^2^
**Unadjusted**									
Life balance	0.53^**^ (0.36, 0.78)	0.10	4.71	0.30^***^ (0.18, 0.52)	0.08	7.49	0.42^***^ (0.28, 0.63)	0.09	7.64
**Adjusted**									
Life balance	0.55^**^ (0.37, 0.82)	0.11	15.05	0.31^***^ (0.18, 0.55)	0.09	4.72	0.42^***^ (0.27, 0.65)	0.09	13.38
Age	1.01 (0.91, 1.12)	0.06			1.12 (0.99, 1.28)		0.07			1.03 (0.92, 1.15)	0.06	
Gender identity^a^										
Women	1.48 (0.63, 3.43)	0.63			0.97 (0.34, 2.73)		0.51			1.18 (0.51, 2.78)	0.52	
Other gender identity	2.48 (0.31, 20.03)	2.64			3.22 (0.29, 35.63)		3.95			1.31 (0.14, 12.08)	1.49	
Race/ethnicity^b^	0.91 (0.40, 2.06)	0.38			0.46 (0.16, 1.26)		0.23			0.63 (0.27, 1.47)	0.27	

CI, confidence intervals; SE, standard error; χ^2^, goodness-of-fit test.

^a^Reference group is men.

^b^Reference group is non-white.

^**^p < 0.01; ^***^p < 0.001.

## 4 Discussion

This is the first US study, and among the first globally, to investigate work-method autonomy, perceived boundary control, life balance, and mood disorder symptoms among postdocs. Nearly half of the postdocs in our study reported symptoms of either anxiety or depressive disorder, which did not vary by demographic characteristics. Work-method autonomy and perceived boundary control were positively associated with life balance. Moreover, postdocs who reported higher life balance were less likely to report mood disorder symptoms.

The rates of anxiety and depressive disorder symptoms reported by postdocs in this sample are higher than those reported among general adult population during the same time interval, both nationally and at the state level (April–July 2022) based on information published by the National Center for Health Statistics (2022). For example, 39.3% of postdocs in our sample reported anxiety disorder symptoms compared with 27.8% of adults nationwide and 26.1% in Massachusetts (National Center for Health Statistics, 2022). Similarly, 26.5% of postdocs in our study reported depressive disorder symptoms compared with 22.3% of adults nationwide and 20.4% in Massachusetts (National Center for Health Statistics, 2022). This may suggest that postdocs face a more significant mental health crisis than the general population, which warrants greater attention to postdocs' mental health needs.

Our findings provide evidence that greater work-method autonomy and perceived boundary control are positively linked to life balance. These findings suggest that recruiting and retaining the postdoc workforce may require a greater emphasis on autonomy and boundary control, allowing postdocs to decide when, where, and how they complete work tasks. Institutions and supervisors should pay attention to promoting healthier work environments for postdocs by respecting postdoc boundaries and autonomy. Providing motivation and flexibility can enhance postdoc autonomy and help them better manage their personal and professional commitments.

Similar to other studies (Deci and Ryan, 2000; Badri and Panatik, 2020), our findings support the hypothesis that life imbalance increases the likelihood of having mood disorder symptoms among postdocs. These findings illustrate the need for institutional support to promote awareness of the potential influences of life balance on the wellbeing of postdocs. For example, institutions can provide postdocs with various workshops and seminars on life balance and mental health, just as they do for undergraduate and graduate students. As postdocs largely work directly with their supervisors, postdoc supervisors can play a vital role in supporting postdocs' life balance and mental health through regular conversations about project timelines, expectations, and what postdocs need to ensure that their scope of work is manageable and realistic. Offering clear guidance on and expectations about workloads can help postdocs maintain a healthy life balance and reduce their mental health risks.

Our study has some limitations. First, we could not reach all Massachusetts postdocs due to the lack of a comprehensive contact list. We only recruited participants who received the survey invitation and those who were interested, possibly leading to selection bias. Second, our sample of 117 postdocs was small, all from Boston. However, the distributions of race/ethnicity, immigration status and study discipline within our sample mimic those of national data and other national studies (McConnell et al., 2018; PostdocInUSA, 2021; National Center for Science Engineering Statistics, 2022), potentially reflecting the true distribution of these characteristics among postdocs across the country. Because institutions in Massachusetts do not readily share their postdocs information, we were unable to assess how our sample compares to the overall postdoc population in Massachusetts. However, small sample sizes are common in postdoc studies (Ysseldyk et al., 2019; Pitt et al., 2021), possibly due to limited survey reach and postdocs' time constraints. Future research should employ collaborative recruitment to engage postdocs more effectively. Third, the use of self-reported measures might have introduced recall and social desirability biases. Fourth, our cross-sectional design limits causal inference. Longitudinal research is needed to explore whether work-method autonomy and perceived boundary control can predict mental health distress related to life balance, which might work as a mediator in this relationship.

## 5 Conclusion

Almost 50% of the postdocs in this study reported having either anxiety or depressive disorder symptoms. High rates of mood disorder symptomology were observed regardless of demographic characteristics. Postdoc mental health appeared to be influenced by life balance which may be enhanced by providing work-method autonomy and increasing perceived boundary control. Combating mental health risks among postdocs may require sustainable systematic changes within academia with the support of institutions, supervisors, and the wider research communities.

## Data Availability

The data that support the findings of this study are available from the corresponding author upon reasonable request.
