# Supplementary material for: Examining the associations between mental health, life balance, work-method autonomy, and perceived boundary control among postdoctoral fellows
Source: Front Psychol. 2024 Dec 19;15:1416724. doi: 10.3389/fpsyg.2024.1416724 (PMC11693439; doi:10.3389/fpsyg.2024.1416724)
Supplement: Supplementary file 1 [file Table_1.DOCX]

**Supplementary Table 1.** Key Variables Descriptive Statistics

| Variables | Mean (SD) | Min | Max | Skew | Kurtosis |
| --- | --- | --- | --- | --- | --- |
| Anxiety disorder symptom^a^ | 2.60 (1.89) | 0 | 6 | 0.55 | -0.80 |
| Depressive disorder symptom^b^ | 1.76 (1.70) | 0 | 6 | 0.85 | -0.04 |
| Work-method autonomy | 3.96 (0.91) | 1 | 5 | -1.27 | 1.73 |
| Boundary control | 3.39 (0.97) | 1 | 5 | -0.64 | 0.05 |
| Life balance | 2.68 (1.07) | 1 | 5 | 0.06 | -1.09 |

*Note.* ^a^Based on the mean GAD-2 score; ^b^Based on PHQ-2 score.
