# Supplementary material for: Examining the associations between mental health, life balance, work-method autonomy, and perceived boundary control among postdoctoral fellows
Source: Front Psychol. 2024 Dec 19;15:1416724. doi: 10.3389/fpsyg.2024.1416724 (PMC11693439; doi:10.3389/fpsyg.2024.1416724)
Supplement: Supplementary file 2 [file Table_2.DOCX]

**Supplementary Table 2.** Correlation Matrix of All Variables

| Variables | (1) | (2) | (3) | (4) | (5) | (6) | (7) | (8) | (9) | (10) | (11) | (12) | (13) | (14) |
| --- | --- | --- | --- | --- | --- | --- | --- | --- | --- | --- | --- | --- | --- | --- |
| (1) Anxiety disorder symptoms | 1.00 |  |  |  |  |  |  |  |  |  |  |  |  |  |
| (2) Depressive disorder symptoms | 0.46^***^ | 1.00 |  |  |  |  |  |  |  |  |  |  |  |  |
| (3) Either anxiety or depressive disorder symptoms | 0.88^***^ | 0.66^***^ | 1.00 |  |  |  |  |  |  |  |  |  |  |  |
| (4) Perceived boundary control | -0.13 | -0.18^*^ | -0.18^*^ | 1.00 |  |  |  |  |  |  |  |  |  |  |
| (5) Job autonomy | 0.03 | -0.26^*^ | -0.11 | 0.22^*^ | 1.00 |  |  |  |  |  |  |  |  |  |
| (6) Life balance | -0.30^*^ | -0.44^*^ | -0.40^*^ | 0.44^*^ | 0.33^*^ | 1.00 |  |  |  |  |  |  |  |  |
| (7) Age | 0.09 | 0.16^*^ | 0.12 | -0.06 | -0.20^*^ | -0.23^*^ | 1.00 |  |  |  |  |  |  |  |
| (8) Gender identity | 0.13 | 0.16 | 0.06 | -0.06 | 0.05 | -0.07 | -0.03 | 1.00 |  |  |  |  |  |  |
| (9) Race/ethnicity | 0.14 | 0.13 | -0.03 | 0.01 | 0.00 | 0.11 | -0.13 | 0.25^**^ | 1.00 |  |  |  |  |  |
| (10) Annual postdoc income | 0.27 | 0.09 | -0.06 | 0.03 | -0.06 | 0.07 | 0.03 | -0.08 | -0.19^*^ | 1.00 |  |  |  |  |
| (11) Parental status | -0.11 | 0.03 | -0.08 | 0.01 | 0.02 | -0.13 | 0.28^*^ | 0.16 | 0.22 | 0.23 | 1.00 |  |  |  |
| (12) Marital status | -0.05 | -0.16 | -0.07 | -0.05 | 0.05 | 0.02 | 0.17 | 0.16 | 0.22 | 0.22 | 0.34 | 1.00 |  |  |
| (13) Discipline | 0.34 | 0.39 | -0.02 | 0.05 | 0.00 | 0.13 | -0.06 | 0.30 | 0.34 | 0.33 | 0.38 | 0.23 | 1.00 |  |
| (14) Immigration status | 0.09 | 0.02 | 0.07 | -0.04 | -0.11 | -0.05 | 0.14 | 0.17 | 0.25 | 0.19 | -0.20 | -0.10 | 0.38 | 1.00 |

*Note.* ^*^ *p* <.05; ^**^*p* <.01; ^***^*p* <.001*.* Pearson’s R was used for the correlation matrix between continuous variables; Spearman’s *Rho* was used for the correlation matrix between continuous and categorical variables; Phi coefficient(φ) was used for the correlation matrix between categorical variables. Continuous variables include perceived boundary control, job autonomy, life balance, and age; Categorical variables include anxiety disorder symptoms, depressive disorder symptoms, either anxiety or depressive disorder symptoms, gender identity, race/ethnicity, annual postdoc income, parental status, marital status, discipline, and immigration status.
